# Study on Thermal Cycling Reliability of Epoxy-Enhanced SAC305 Solder Joint

**DOI:** 10.3390/polym16182597

**Published:** 2024-09-14

**Authors:** Peng Zhang, Songbai Xue, Lu Liu, Jianhao Wang, Hiroaki Tatsumi, Hiroshi Nishikawa

**Affiliations:** 1 College of Materials Science and Technology, Nanjing University of Aeronautics and Astronautics, Nanjing 210016, China; 2 Joining and Welding Research Institute, Osaka University, Ibaraki 5670047, Japan; 3 Institute of Intelligent Welding and Precision Manufacturing, Shanghai Jiao Tong University, Shanghai 200240, China

**Keywords:** SAC305 composite solder joint, epoxy, thermal cycling, microstructure, shear force, fracture morphology

## Abstract

In this work, epoxy was added into commercial Sn-3.0Ag-0.5Cu (SAC305) solder paste to enhance the thermal cycling reliability of the joint. The microstructure and fracture surface were observed using a scanning electron microscope/energy dispersive spectrometer (SEM/EDS), and a shear test was performed on the thermally cycled joint samples. The results indicated that during the thermal cycling test, the epoxy protective layer on the surface of the epoxy-enhanced SAC305 solder joint could significantly alleviate the thermal stress caused by coefficients of thermal expansion (CTE) mismatch, resulting in fewer structural defects. The interfacial compound of the original SAC305 solder joints gradually coarsened due to the accelerated atomic diffusion, but epoxy-enhanced SAC305 solder joints demonstrated a thinner interfacial layer and a smaller IMC grain size. Due to the reduced stress concentration and the additional mechanical support provided by the cured epoxy layer, epoxy-enhanced SAC305 solder joints displayed superior shear performance compared to the original joint during the thermal cycling test. After 1000 thermal cycles, Cu-Sn IMC regions were observed on the fracture surfaces of the original SAC305 solder joint, exhibiting brittle fracture characteristics. However, the fracture of the SAC305 solder joint with 8 wt.% epoxy remained within the solder bulk and exhibited a ductile fracture mode. This work indicates that epoxy-enhanced SAC305 solder pastes display high thermal cycling reliability and could meet the design needs of advanced packaging technology for high-performance electronic packaging materials.

## 1. Introduction

In recent years, with the rapid development of electronic information technology, electronic products characterized by small sizes and fabulous performance have gained widespread popularity. To further extend Moore’s law, numerous high-density packaging technologies, such as three-dimensional (3D) packaging, were proposed to increase heterogeneous integration and input/output (I/O) density [1]. Within the packaging structure, solder joints play a crucial role, serving not only as electrical and mechanical connections between components but also as essential channels for heat dissipation [2,3].

In advanced packaging structures, the dramatic reduction in micro-joint size and the increase in packing density can give rise to serious Joule heating problems. Throughout the service life of electronic products, solder joints are inevitably affected by fluctuations in temperature load. Given the significant mismatch among the coefficients of thermal expansion (CTE) of various components, solder joints undergo alternating thermal stress and strain during temperature cycles, posing a serious threat to the reliability of solder joints [4,5]. Generally, a thin and continuous intermetallic compound (IMC) layer forms between molten solder and the substrate during the reflow process, indicating a robust metallurgical bond [6]. However, due to the intrinsic brittleness of the interfacial IMC layer, its excessive growth during subsequent thermal treatments will seriously diminish the interfacial strength and overall joint reliability [7]. Particularly during thermal cycling, stress concentration typically occurs near the interface region, where cracks are more likely to nucleate and propagate, resulting in the thermal fatigue fracture of solder joints [8].

As electronic devices continue to undergo miniaturization, current solder alloy systems can no longer meet the required thermal reliability during service. Consequently, significant efforts have been devoted to developing novel composite solder materials with improved performance [9,10]. Among these, composite solder alloys doped with microparticles or nanoparticles have demonstrated remarkable comprehensive properties, particularly in terms of mechanical properties and reliability [11,12,13]. For instance, Illés et al. [14] discovered that adding TiO_2_ nanoparticles to SAC305 solder alloy could refine Cu_6_Sn_5_ and Ag_3_Sn IMC in the solder bulk. This addition also hindered the movement of dislocations under external load, leading to a higher hardness of the composite solder. Furthermore, TiO_2_ NPs bonded to the Sn atoms through the O atom of TiO_2_, forming a stable TiO_2_-Sn structure that was resistant to further oxidation. As a result, the composite solder exhibited significant corrosion resistance [15]. Nishikawa et al. [16] investigated the enhancement effect of NiO-modified ZnO (NiO/ZnO) particles on the mechanical properties of Sn1.0Ag0.5Cu solder. They found that the strength and ductility of the composite solder were improved simultaneously due to the load transfer, grain refinement, and misfit dislocation effects. It has been reported that doping SiC nanowires to pure Sn solder joints could effectively retard atomic diffusion and inhibit IMC growth during thermal cycling, owing to the pinning effect and adsorption theory [17]. Adding Mo NPs could increase the activation energy of atomic diffusion and reduce the diffusion coefficient of the SAC305 solder joint during the thermal cycling test, resulting in a slower growth rate of the IMC layer [18]. Due to the thin IMC layer and the inhibitory effect of Mo nanoparticles on dislocation motion, the composite solder joint displayed superior tensile properties than the original solder joint. However, due to the small size effect and high surface energy, NPs are prone to agglomeration, making it challenging to achieve a uniform distribution within solder joints [19]. In addition, the complex preparation process and high cost also severely restrict their application in solder modification.

Due to its low cost and straightforward manufacturing process, lead-free solder materials containing epoxy have received intensive attention. The epoxy addition notably improves the spreadability of solder pastes, and the mechanical properties of the epoxy-containing joints are enhanced by the blocking effect of the cured epoxy fillet [20,21]. Jung et al. [22] revealed that, compared to the original Sn-58Bi solder joint, the Sn-58Bi epoxy solder joint exhibited a slower IMC growth rate and higher shear strength during aging treatment. Moreover, the Sn-58Bi epoxy solder joint also demonstrated superior thermal shock resistance, attributed to the additional bonding area provided by the epoxy [23]. Sharma et al. [24] discovered that the epoxy layer could act as a stress reliever, thereby impeding the growth of interfacial IMC during a thermal shock test. Our group also conducted thermal reliability tests on epoxy-containing Sn-58Bi solder joints [25]. Remarkably, even after 1000 cycles of thermal cycling or 1000 h of high temperature and high humidity tests, the epoxy layer continued to enhance the mechanical properties of the Sn-58Bi solder joints. 

At present, numerous research achievements have been made in the preparation and performance characterization of epoxy-containing low-temperature Sn-Bi solder paste. However, there are few reports on the influence of epoxy addition on the mechanical properties and reliability of Sn-Ag-Cu solder paste, which is widely used in the reflow soldering process of the electronic packaging industry due to its excellent wettability and thermal reliability. To meet the growing demand for high-performance soldering materials in the electronics packaging industry, relevant research is of huge significance. In our previous study, novel epoxy-enhanced Sn-3.0Ag-0.5Cu (SAC305E) solder pastes were developed and exhibited fabulous spreadability and high shear performance [26,27]. To ensure the long-term service reliability of epoxy-enhanced SAC305 solder pastes, in this work, the effect of thermal cycling on the microstructure evolution, interfacial behavior, and mechanical properties of SAC305E solder joints were investigated. The damage mechanism and failure behavior of the original SAC305 and SAC305E solder joints under the alternating thermal load were compared and the influence mechanism of epoxy addition was further discussed.

## 2. Materials and Methods

In this work, commercial SAC305 solder paste containing no-clean flux and SAC305 solder powders with a diameter of 25–45 μm were used as the original condition. The epoxy curing solution contained E51 epoxy resin, an anhydride curing agent, and an accelerator with a ratio of 100:85:0.5–3. SAC305E solder pastes were prepared through mechanical mixing of the original SAC305 solder pastes and E51 epoxy curing solution by a solder paste mixer (MIX500D2, RobotDigg, Shanghai, China). The mixing process was conducted at 600 rpm for 5 min to uniformly disperse the epoxy curing solution. In addition, epoxy-enhanced SAC305 solder paste containing x% (weight percent) epoxy curing solution was labeled as SAC305E-x solder paste. In our previous study, it was found that only when the epoxy content reached 8%, a complete resin layer could be formed on the surface of the solder joint. However, excessive epoxy addition (over 12 wt%) led to a void formation in the composite solder joint [26]. Therefore, in this work, SAC305E-8 and SAC305E-12 solder pastes were selected for further investigation. 

**Figure 1:**
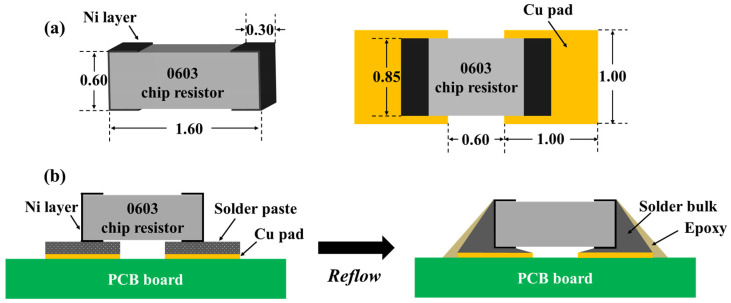
Schematic diagram of (**a**) The dimensions of the chip resistor and Cu pad, and (**b**) the preparation of the SAC305E solder joint.

**Figure 2:**
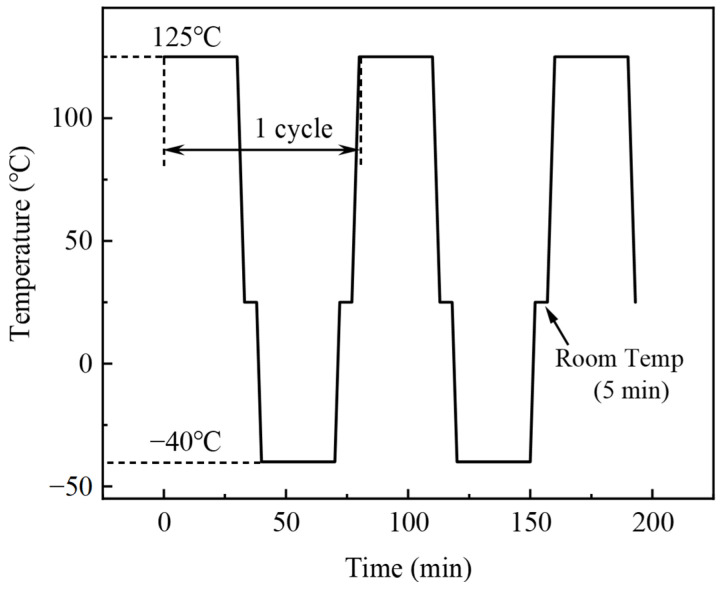
Temperature profile of the thermal cycling test.

Subsequently, SAC305E solder pastes were printed to the Cu pad on the printed circuit board (PCB) by stencil printing, and then 0603 chip resistors were soldered to the Cu pads by reflow soldering. The dimensions of the chip resistor and Cu pad used in this work are displayed in Figure 1a. The peak temperature of the reflow process was 250 °C with a total reflow time of 5 min. During the reflow, the solder alloy melted and wetted the Ni layer of the chip resistor and Cu pad, forming a metallurgical bonding. At the same time, the epoxy floated onto the joint surface and cured, as shown in Figure 1b. Board-level thermal cycling test was carried out by using thermal shock test chambers (TSA-43EL, Espec, Osaka, Japan). The as-reflowed samples were subjected to a thermal profile ranging from −40 to 125 °C for 250, 500, 750, and 1000 cycles. Both the high and low-temperature storage times were set to 30 min, with the room temperature residence time set to 5 min, as shown in Figure 2. As a control, the original SAC305 solder joint was prepared using the same reflow process and subjected to the same thermal cycling test.

**Figure 3:**
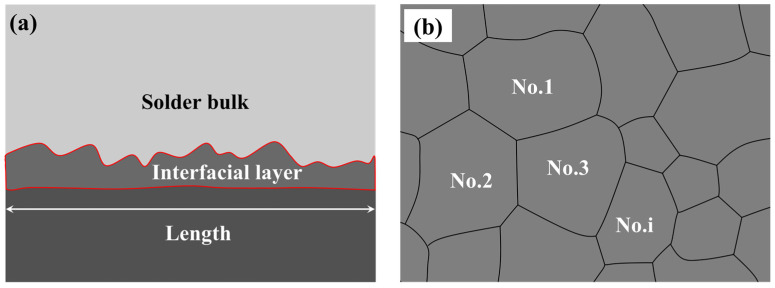
Schematic diagram of measuring (**a**) IMC thickness and (**b**) IMC grain size.

The macroscopic morphology and cross-sectional microstructure of the solder joints were examined using a scanning electron microscope (SEM, JSM-IT200, JEOL, Tokyo, Japan), and the corresponding elemental composition analysis was analyzed by energy dispersive spectroscopy (EDS). Following the sandpaper grinding and polishing process, the cross-sectional microstructure of the joint was observed. To enhance the visibility of top-view morphologies of the interfacial compounds, the solder bulk above the interfacial layer was selectively removed using thin nitric acid. The thickness and grain size of the interfacial compound were measured and calculated with the aid of Image J software (version: 1.54d). The schematic diagram of measuring IMC thickness and IMC grain size was shown in Figure 3 and was calculated by Equations (1) and (2), respectively.

(1) $$X=\frac{A}{L}$$

(2) $$S=\frac{\sum_{1}^{n}S_{i}}{n}$$

where *X* is the IMC thickness, *A* is the area of the measured IMC layer, *L* is the length of the measured IMC layer, *S* is the average IMC grain size, *n* is the total number of IMC grains in the observed zone, and *S_i_* is the area of No. *i* IMC grain. At the same parameter, the average values of interfacial layer thickness and IMC grain size at five random positions in the solder joint were calculated as the final result.

To investigate the mechanical properties of the joints during the thermal cycling test, their shear forces were measured using a bonding test machine (PTR1102, Rhesca, Tokyo, Japan) in accordance with Japanese Industrial Standard JIS Z3198-7 [28]. The shear tip height was set at 100 μm with a forward speed of 2 mm/min. For each condition, 10 chip resistor solder joints were tested, and their average value was noted. After the shear test, the fracture morphologies were further analyzed and observed by SEM and EDS.

## 3. Results

### 3.1. Macro and Cross-Sectional Structure of the Joint

**Figure 4:**
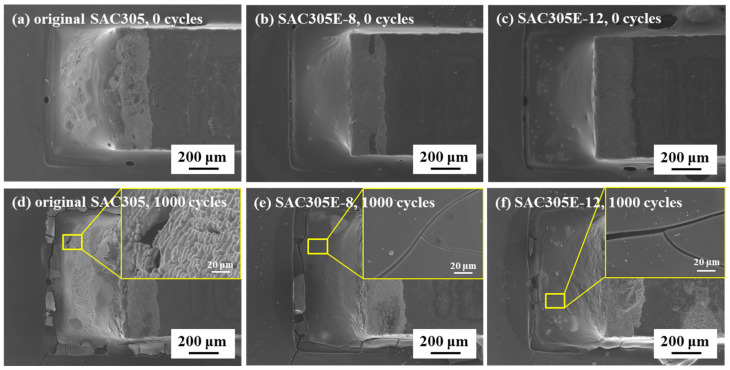
Macroscopic surface of the solder joints after (**a**–**c**) 0 cycles and (**d**–**f**) 1000 cycles: (**a**,**d**) original SAC305; (**b**,**e**) SAC305E-8; (**c**,**f**) SAC305E-12.

Figure 4 exhibits the macroscopic surface of the original SAC305 and epoxy-enhanced SAC305 solder joints during the thermal cycling test. In Figure 4a, it could be observed that after reflow soldering (i.e., 0 cycles), some flux residue remained on the surface of the original SAC305 solder joint. However, with the incorporation of 8% and 12% epoxy into SAC305 solder paste, cured epoxy protective layers appeared on the joint surfaces, as shown in Figure 4b,c. During reflow soldering, after the solder powder in SAC305E solder pastes melted, due to the incompatibility between epoxy and the molten solder alloy, epoxy gradually overflowed due to its low density and covered the surface of the solder joint and gradually solidified. After the solder joint was cooled and solidified, a complete and even protective layer of epoxy was formed on the surface of the solder joint. After 1000 thermal cycles, noticeable gully areas were detected on the joint surface, as presented in Figure 4d. This may be attributed to the thermal stress caused by the alternating temperature load. In contrast, for the epoxy-enhanced SAC305 solder joints, the formation of an epoxy-covered layer resulted in a relatively flat joint surface, with only a few elongated cracks found in the epoxy layer, as demonstrated in Figure 4e,f.

**Figure 5:**
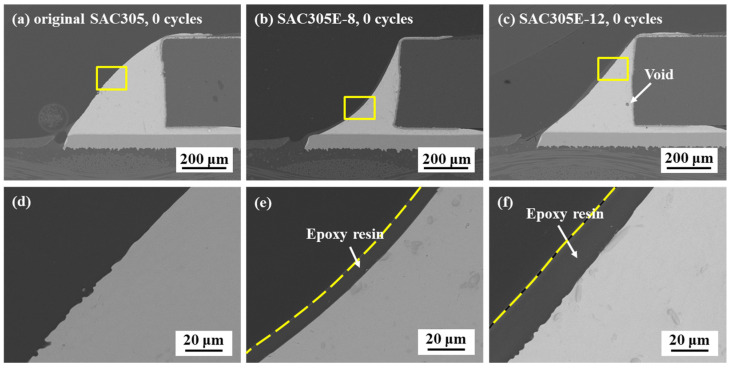
Cross-sectional microstructure of the solder joints after 0 cycles: (**a**) original SAC305; (**b**) SAC305E-8; (**c**) SAC305E-12; (**d**–**f**) magnified images of the marked regions in (**a**–**c**).

Figure 5 shows the cross-sectional microstructure of the original SAC305 and epoxy-enhanced SAC305 solder joints after reflow. It is evident from Figure 5e,f that the cured epoxy layer adhered closely to the joint surface, forming fillets with the same shape as the solder bulk. However, when the epoxy content reached 12%, excessive organic components, including epoxy and flux, were challenging to escape from the molten solder alloy during reflow. This led to their retention in the solder bulk, resulting in the formation of voids. Ohno et al. [29] pointed out that the presence of voids harmed the fatigue life of the solder joints and this effect became more apparent as the void ratio increased. Therefore, the epoxy content needs to be strictly controlled. It should also be noticed that with the addition of epoxy, the fillet shapes of the solder joints transformed from convex-shape to concave-shape, as shown in Figure 5a–c. This phenomenon could be attributed to the ratio reduction of solder powder in the solder paste with the incorporation of epoxy. In surface mounted technology (SMT), the volume of solder paste printed onto the metal pads is fixed due to the consistent aperture and thickness of the metal mask. In this work, with the increased epoxy content, the volume of SAC305 solder alloy gradually decreased, leading to the fillet shape evaluation. Generally, it is considered that the chip resistor solder joint bearing a tiny concave fillet shape displayed a longer thermal fatigue life than those with other fillet shapes [29,30]. As a result, the fillet shape caused by the epoxy addition may help to enhance the thermal cycling reliability of the SAC305 solder joints.

**Figure 6:**
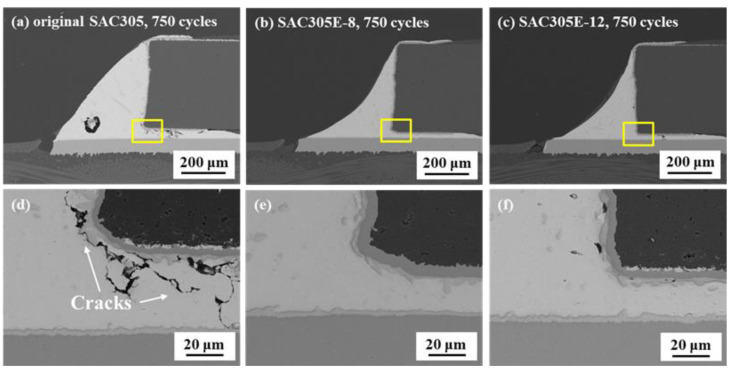
Cross-sectional microstructure of the solder joints after 750 cycles: (**a**) original SAC305; (**b**) SAC305E-8; (**c**) SAC305E-12; (**d**–**f**) magnified images of the marked regions in (**a**–**c**).

After 750 cycles, numerous cracks were detected in the solder bulk between the chip resistor and the Cu substrate in the original SAC305 solder joint, as revealed in Figure 6a,d. This indicates that thermal stress concentration occurred in this region due to the CTE mismatch among the ceramic resistor, solder bulk, IMC layer, and Cu pad. When the accelerated thermal stress and strain exceeded the fracture toughness of the solder bulk, the crack began to initiate. Nevertheless, few structural defects could be observed around the resistor edge in the epoxy-enhanced SAC305 solder joints, as shown in Figure 6b,c,e,f.

**Figure 7:**
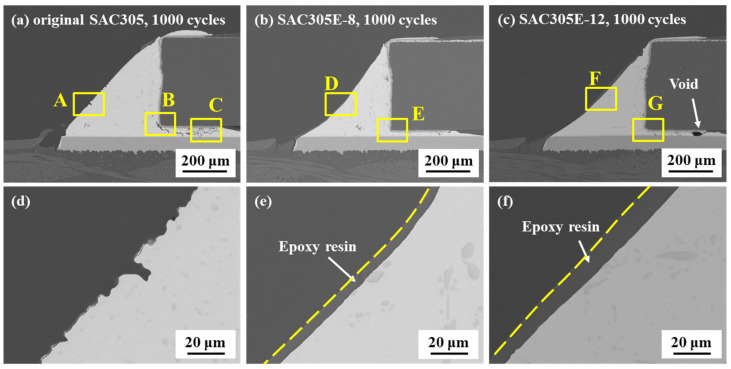
Cross-sectional microstructure of the solder joints after 1000 cycles: (**a**) original SAC305; (**b**) SAC305E-8; (**c**) SAC305E-12; (**d**) magnified image of region A in (**a**); (**e**) magnified image of region D in (**b**); (**f**) magnified image of region F in (**c**).

**Figure 8:**
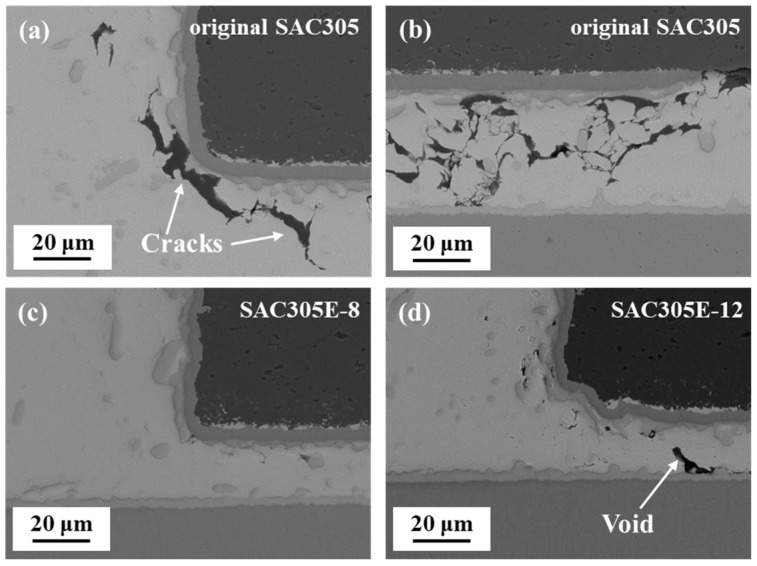
Enlarged SEM images of the marked area in Figure 7: (**a**) region B in Figure 7a; (**b**) region C in Figure 7a; (**c**) region E in Figure 7b; (**d**) region G in Figure 7c.

Figure 7 shows the cross-sectional microstructure of the original SAC305 and epoxy-enhanced SAC305 solder joints after 1000 cycles. It could be seen from Figure 7d that after being subjected to long-term thermal loads, the inclined slope of the original joint became rugged, corresponding to the top-view morphology in Figure 4d. Figure 8 presents the high-magnification images of the marked areas in Figure 7. In Figure 8a,b, numerous cracks were detected between the resistor and the solder/IMC interface which were interconnected, and their propagation path passed through the IMC particles in the solder bulk. As shown in Figure 7e,f, for SAC305 solder joints incorporated with 8% and 12% epoxy, the solder joint fillets remained completely covered by the epoxy layers, maintaining flat appearances even after 1000 cycles. Furthermore, compared to the original SAC305 solder joint, fewer cracks were formed at the resistor edge with the epoxy addition, as revealed in Figure 8c,d. The epoxy protective layer, with its high hardness, is believed to offer mechanical support external to the solder joint. Consequently, it disperses thermal stress, functioning similarly to the filling adhesive (underfill) in BGA packaging structure [24,30]. Therefore, in the epoxy-enhanced SAC305 solder joint, the reduction in thermal stress concentration led to an improvement in thermal resistance.

### 3.2. Interfacial Microstructure

**Figure 9:**
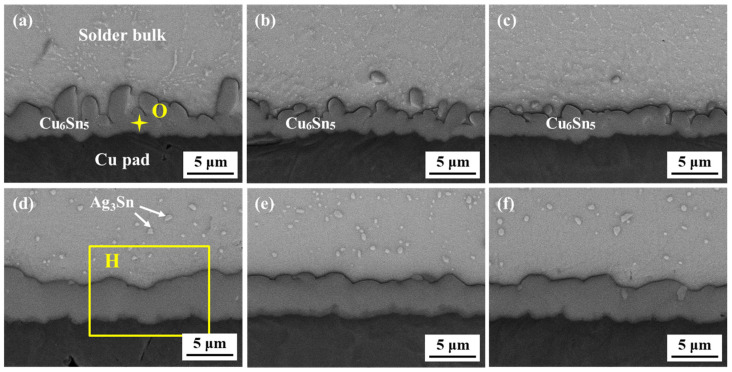
Interfacial microstructure of the solder joints after (**a**–**c**) 0 cycles and (**d**–**f**) 1000 cycles: (**a**,**d**) original SAC305, (**b**,**e**) SAC305E-8, (**c**,**f**) SAC305E-12.

Figure 9 presents the interfacial microstructure of the original SAC305 solder joint and epoxy-enhanced SAC305 solder joints during thermal cycling. It can be observed from Figure 9a–c that a continuous scallop-like IMC layer formed between the solder bulk and the Cu pad in all as-reflowed solder joints. Nevertheless, epoxy-enhanced SAC305 solder joints exhibited better thickness uniformity. EDS analysis of Point O in Table 1 demonstrates that the atomic ratio of Cu and Sn is close to 6:5. According to numerous studies about the interfacial reaction between the lead-free solder and Cu substrate, this interfacial compound could be identified as Cu_6_Sn_5_ [31,32].

**Figure 10:**
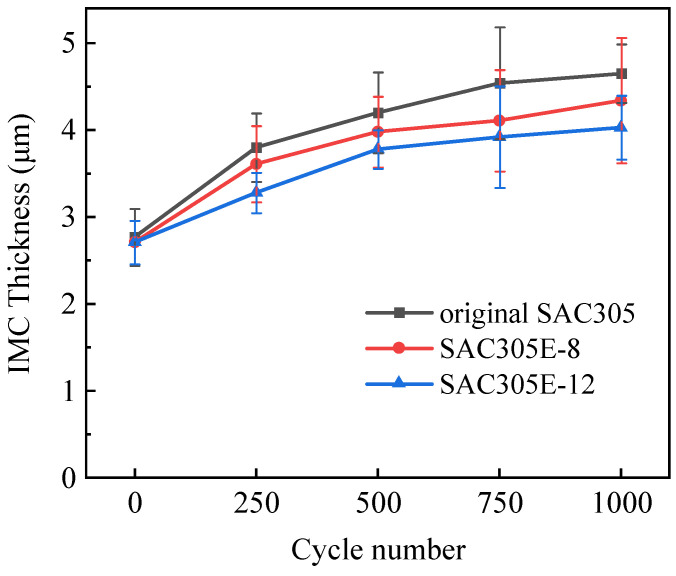
Total thicknesses of interfacial IMC of the solder joints during thermal cycling.

**Figure 11:**
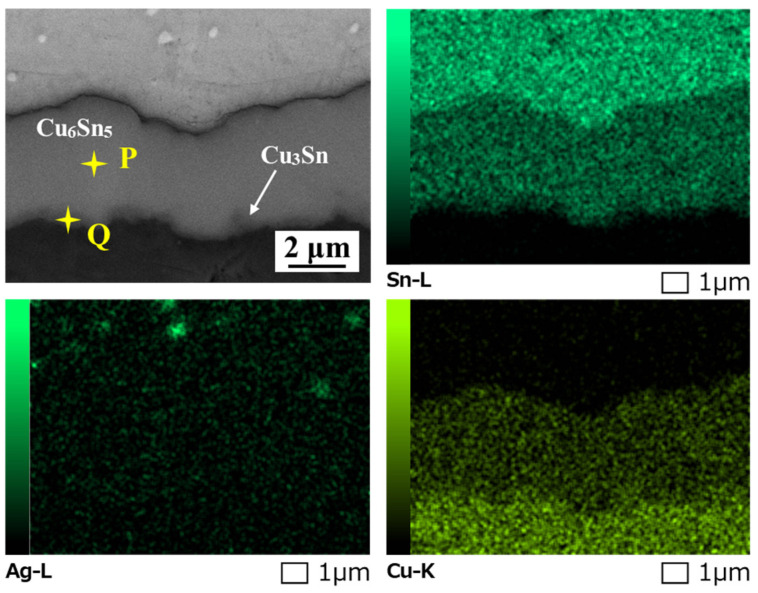
Enlarged SEM image and EDS elemental mapping of region H in Figure 9.

Figure 10 describes the thickness variation of the interfacial layer of the solder joint with thermal cycles. The thickness of the IMC layer in all as-reflowed solder joints remained nearly constant, regardless of the epoxy addition. The growth of the interface layer during reflow is primarily determined by the reflow temperature and reflow time [33]. Since all samples underwent the same reflow process, the addition of epoxy had minimal impact on the overall thickness of the interface layer. After 1000 cycles, the interfacial layers of all joints thickened and exhibited a bilayer structure, as shown in Figure 9d–f. Figure 11 presents the magnified image and EDS elemental mapping of region H in Figure 9d. A new thin IMC formed between Cu_6_Sn_5_ (such as Point P) and Cu substrate and displayed less Sn concentration than Cu_6_Sn_5_ IMC. Combined with the EDS analysis of Point Q, it could be inferred as Cu_3_Sn compound, which is consistent with the research of Lin [34] and Sun [35]. After 1000 cycles, the total IMC thicknesses of the SAC305E-8 and SAC305E-12 solder joints were 4.0 μm and 4.2 μm, respectively, which were smaller than that of the original joint at 4.7 μm. During the high-temperature stage, the atomic diffusion near the joint interface was accelerated, resulting in the thickened morphology of the interfacial layer. In addition, the microstructure of the solder bulk in all solder joints coarsened after 1000 cycles, and the size of Ag_3_Sn IMC particles near the interfacial layer obviously increased. The morphology evolution of Ag_3_Sn particles would obviously reduce their dispersion-strengthening effect, resulting in a significant decrease in the strength of the solder bulk.

**Table 1:** EDS analysis results of the marked points in Figure 9 and Figure 11 (at%).

Point	Cu	Sn
O	56.94	43.06
P	53.26	46.74
Q	74.38	25.62

**Figure 12:**
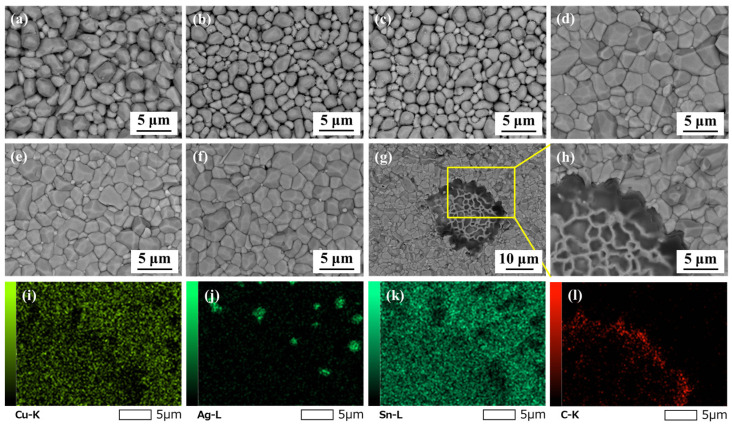
Top-view morphologies of interfacial layers in the solder joints after (**a**–**c**) 0 cycles and (**d**–**h**) 1000 cycles: (**a**,**d**) original SAC305; (**b**,**e**) SAC305E-8; (**c**,**f**–**h**) SAC305E-12; (**i**–**l**) EDS elemental mapping of (**h**).

**Figure 13:**
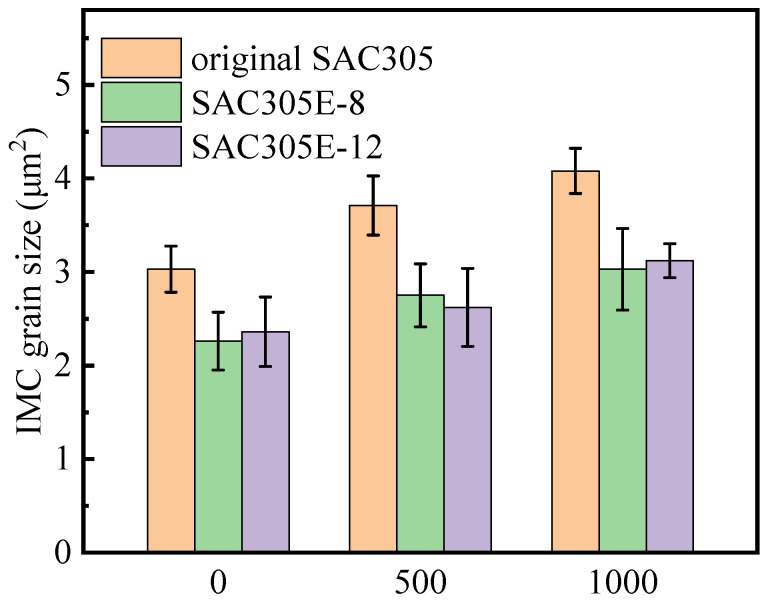
Grain sizes of interfacial IMCs of the solder joints during thermal cycling.

**Figure 14:**
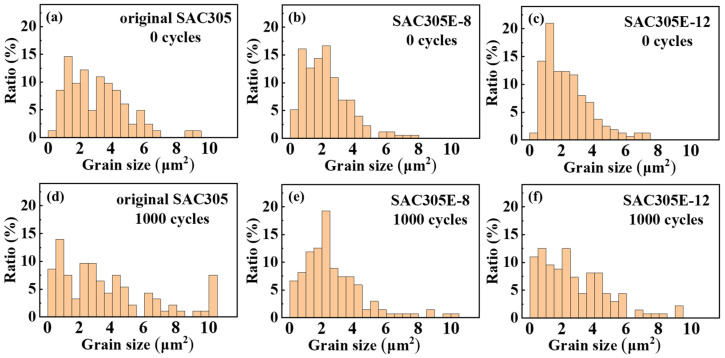
Grain size distribution of interfacial IMCs in Figure 12 after (**a**–**c**) 0 cycles and (**d**–**f**) 1000 cycles: (**a**,**d**) original SAC305; (**b**,**e**) SAC305E-8; (**c**,**f**) SAC305E-12.

Figure 12 depicts the top-view morphologies of the interfacial layers after removing the above solder bulk by acid etching. As shown in Figure 12a–c, the interfacial Cu_6_Sn_5_ IMC of the as-reflowed joints displayed rough morphologies and obvious gaps between Cu_6_Sn_5_ particles. Figure 13 displays the corresponding grain sizes of the interfacial layers in the original SAC305 and SAC305E solder joints. After reflow soldering, the grain size of interfacial Cu_6_Sn_5_ IMC in the original SAC305, SAC305E-8, and SAC305-12E solder joints were 3.0 μm, 2.3 μm, and 2.4 μm, respectively. It can be seen that the grain size of IMC particles in the as-reflowed joint was notably reduced with the epoxy addition. Figure 14 displayed the grain size distribution of the interfacial IMC before and after the thermal cycling test (the statistical data came from Figure 12). Compared to the original SAC305 solder joint, the as-reflowed SAC305E-8 and SAC305E-12 solder joints demonstrated more uniform grain sizes of the interfacial IMC, predominantly concentrated within the range of 0–4 μm^2^. 

During reflow soldering, when molten Sn-based solder alloy comes into contact with the Cu substrate, the Cu atoms from the Cu substrate quickly dissolve into Sn, and the solder composition near the interface between the liquid solder alloy and Cu substrate quickly reaches a metastable solubility [32]. Due to the strong driving force of the chemical reaction between Cu and Sn atoms at the metastable composition, Cu_6_Sn_5_ grains can be rapidly formed through non-uniform nucleation and growth at the interface of liquid solder alloy/Cu substrate [31]. Galiano et al. [36] investigated the nucleation kinetics of Cu_6_Sn_5_ IMC formed by the interfacial reaction between molten Sn and Cu substrate in a short period (1 s and 2 s). The results showed the nucleation driving force of Cu_6_Sn_5_ IMC decreased continuously with increasing reaction temperature. When the reaction temperature was below 260 °C, the number of Cu_6_Sn_5_ grains generated per unit area gradually increased with the increase in reaction temperature, but the Cu_6_Sn_5_ grain radius continuously decreased. In this work, the exothermic curing reaction of epoxy occurred before the melting of the solder alloy. When the SAC305 solder alloy powder in the SAC305E solder paste began to melt, there was still a large amount of epoxy around the interface of the solder alloy/Cu substrate, and the heat generated by the curing reaction increased the temperature near the interface area. Therefore, in the early stage of the interfacial reaction, the temperature at the interface of the SAC305E solder joint was higher than that of the original SAC305 solder joint, so the SAC305E solder joint had lower Cu_6_Sn_5_ nucleation driving force and higher Cu_6_Sn_5_ nucleation rate, and thus Cu_6_Sn_5_ grains were finer (as shown in Figure 13). As the epoxy overflowed from the liquid solder alloy, its curing reaction no longer had an obvious effect on the growth of interfacial IMC. However, the kinetics and thermodynamics of epoxy in reflow soldering need to be further verified. 

After 1000 thermal cycles, significant grain coarsening occurred near the IMC particle gap in the ripening process, resulting in the flat cross-sectional microstructure and smooth top-view morphologies, as exhibited in Figure 9d–f and Figure 12d–f. As shown in Figure 13, after 1000 cycles, the IMC grain sizes of the original SAC305 solder joint increased to 4.1 μm. However, the IMC grain sizes of the SAC305E-8 and SAC305E-12 solder joints were 3.0 μm and 3.1 μm, respectively, which were 26.8% and 24.4% smaller than that of the original SAC305 solder joint. As presented in Figure 14d–f, the proportion of IMC grain size exceeding 6 μm^2^ in the original joint was higher compared to the epoxy-enhanced joints. This means that the interfacial compound of the original solder joint was more severely coarsened. In addition, some black epoxy residue was observed in the top-view image of the SAC305E-12 solder joint after 1000 cycles, which was cured during reflow soldering and still adhered to the interfacial layer after thermal cycling, as shown in Figure 12g–l. The cured organic product disrupted the interface uniformity, which may cause the stress concentration near the interfacial IMC layer due to its inherent brittleness. However, few cracks were detected in the SAC305E-12 solder joints even after 1000 cycles, which indicated that the stress concentration near the joint interface was insufficient to cause crack initiation.

**Figure 15:**
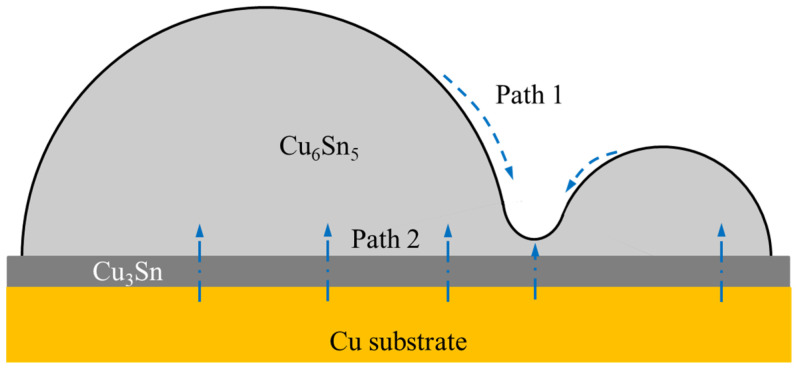
Schematic diagram of Cu diffusion during thermal cycling.

In the solid-state thermal treatment, the atomic diffusion is accelerated and the diffusion of the Cu atom dominates the main role in the Cu/Sn diffusion couple [1]. As illustrated in Figure 15, the Cu atomic flux involved in the interfacial reaction includes two parts. Diffusion path 1 from the peak of the Cu_6_Sn_5_ scallop to the valley is caused by the curvature effect to reduce the surface energy of the scallop-like Cu_6_Sn_5_ grain. Diffusion path 2 from the Cu substrate to the solder bulk is driven by the interfacial reaction to form IMC [37]. Due to the curvature effect and short diffusion path, the growth rate of IMC at the scallop bottom is faster than that at the scallop peak, resulting in a transition of IMC morphology from scallop-type to layer-type. In this work, after reflow soldering, SAC305E solder joints exhibited finer and flatter interfacial IMC morphologies than the original SAC305 solder joint, as displayed in Figure 9a–c and Figure 12a–c. Therefore, near the interface area of the SAC305E solder joint, the driving force of Cu atomic diffusion caused by the curvature effect was reduced, resulting in a lower IMC growth rate during thermal cycling. According to the research results from Han et al. [38], the thermally cycled stress could act as a driving force to promote atomic diffusion, resulting in a higher growth rate of the interfacial IMC. As epoxy can alleviate thermal stress caused by the alternating temperature, the growth of the interfacial IMC in the epoxy-enhanced joints was impeded.

### 3.3. Shear Test

**Figure 16:**
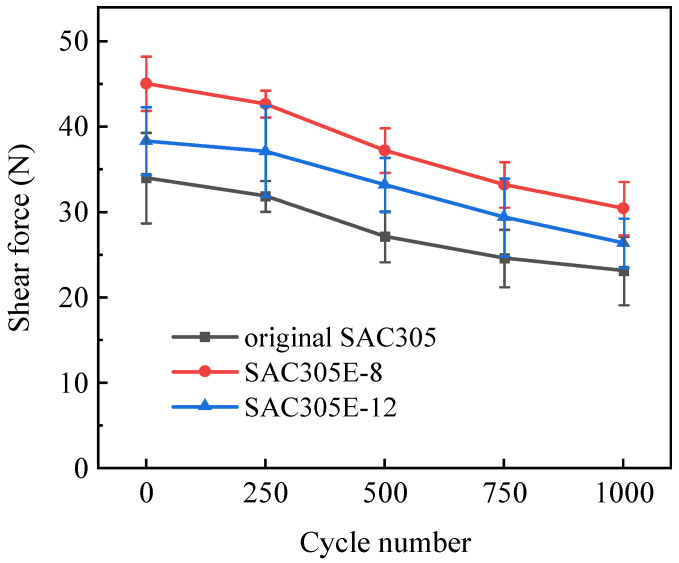
Shear forces of the solder joints with cycle number.

To estimate the damage caused by alternating thermal-mechanical loads on the joint reliability, a shear test was performed every 250 cycles. Figure 16 shows the variation trend of the shear forces of the original SAC305 and SAC305E solder joints during the thermal cycling test. After reflow soldering, SAC305E solder joints displayed higher shear forces than the original SAC305 solder joint because of the mechanical support effect of the cured epoxy layer. However, with the excessive addition of epoxy, void defects appeared in SAC305E-12 solder joints (as depicted in Figure 5), and the shear force of the solder joints decreased instead. It can be concluded that with the increase in thermal cycles, the shear forces of all solder joints exhibited a decreasing trend. After being subjected to 1000 cycles, the shear forces of SAC305E-8 and SAC305E-12 solder joints decreased to 30.5 N and 26.4 N, respectively, which were 31.5% and 13.8% higher than those of the original SAC305 solder joint (23.2 N). According to the results of microstructural analysis, during the thermal cycling test, the microstructure of solder bulk and interfacial IMC in all solder joints gradually coarsened. At the same time, due to the significant CTE difference between different materials in the solder joint, the internal alternating stress and strain gradually accumulated under long-term alternating thermal load [39]. Since the interfacial IMC was generally hard and brittle, the IMC layer thickened continuously, resulting in more severe stress concentration in the solder bulk near the interfacial layer [40]. Therefore, with the increase in the number of thermal cycles, both the original SAC305 solder joint and SAC305 solder joints showed a decreasing trend.

It is evident that epoxy-enhanced SAC305 solder joints bore higher shear forces than the original joint throughout the entire thermal cycling test. The enhancement mechanism of thermal cycling reliability of SAC305E solder joints by adding epoxy could be attributed to the stress release and mechanical support provided by the epoxy layer, as well as the thinner interfacial layer thicknesses of SAC305E solder joints. Firstly, it is widely known that the primary factors affecting the thermal fatigue lifetime of micro-joints subjected to long-term thermal cycling are the accumulated thermal stress and strain [41]. In this work, for the original SAC305 solder joint, numerous thermal fatigue cracks were detected inside the solder bulk close to the resistor and the solder/Cu interface after 750 cycles (as shown in Figure 6), where sufficient thermal stress and strain were accumulated. This can be attributed to the significant CTE mismatch among the chip resistor (ceramics), solder bulk, IMC layer, and Cu pad. The presence of an epoxy layer on the joint surface could release the thermal stress induced by periodic temperature changes, remarkably inhibiting crack initiation and propagation. This reduced the stress concentration within the SAC305E solder joint and enhanced its thermal stability. Secondly, the excessive growth of interfacial IMC during the thermal cycling test significantly impacts joint reliability because the coarsened IMC could cause brittle fracture [42]. Considering the morphology evolution in the interfacial layer, the SAC305E solder joint exhibited a slower IMC growth rate compared to the original joint. This reduction in the thickness of the interfacial layer contributed to a decrease in stress concentration near the interface, mitigating the impact of the inherent brittleness of the interfacial compounds. Finally, the epoxy layer with a high hardness on the surface of SAC305E solder joints could provide obvious mechanical support before and after the thermal cycling test. For the pure cured E51 epoxy, the ultimate tensile strength and the maximum shear strength were 82.0 MPa [43] and 16.7 MPa [44], respectively. When the joint was subjected to the shear load, the epoxy protective layer could act as an extra bonding area, providing extrinsic toughening. Therefore, the epoxy layer surrounding the solder joint not only provided thermal stress dissipation but also mechanical reinforcement. However, it should be noticed that with the prolonged thermal cycling, some cracks were initiated in the cured epoxy, which may diminish the mechanical reinforcement provided by the epoxy protective layer to some extent.

Table 2 lists some composite Sn-Ag-Cu solder joints reinforced by NPs. Similar to nanoparticle reinforcement, adding epoxy into SAC305 solder paste also has a significant improvement effect on the mechanical properties of the solder joints during thermal cycling. It is worth noting that the fabrication process of epoxy-enhanced SAC305 solder paste is much simpler than that of NPs-reinforced solder alloys. In this work, epoxy-enhanced SAC305 solder paste was prepared only by mechanical mixing in an air environment. However, due to the risk of oxidation and aggregation of NPs, high atmosphere requirements (such as inert atmosphere) are usually required during the preparation of NPs-reinforced solder alloy [17,38]. In addition, due to the lower cost of epoxy, epoxy-enhanced SAC305 solder paste has more excellent economic benefits. The preparation process and procedure of the epoxy-enhanced SAC305 solder joint are the same as those of the original SAC305 solder joint. Therefore, in practical use, the epoxy-enhanced SAC305 solder paste prepared in this work will not increase process costs and will not affect production efficiency.

### 3.4. Fracture Morphology

**Figure 17:**
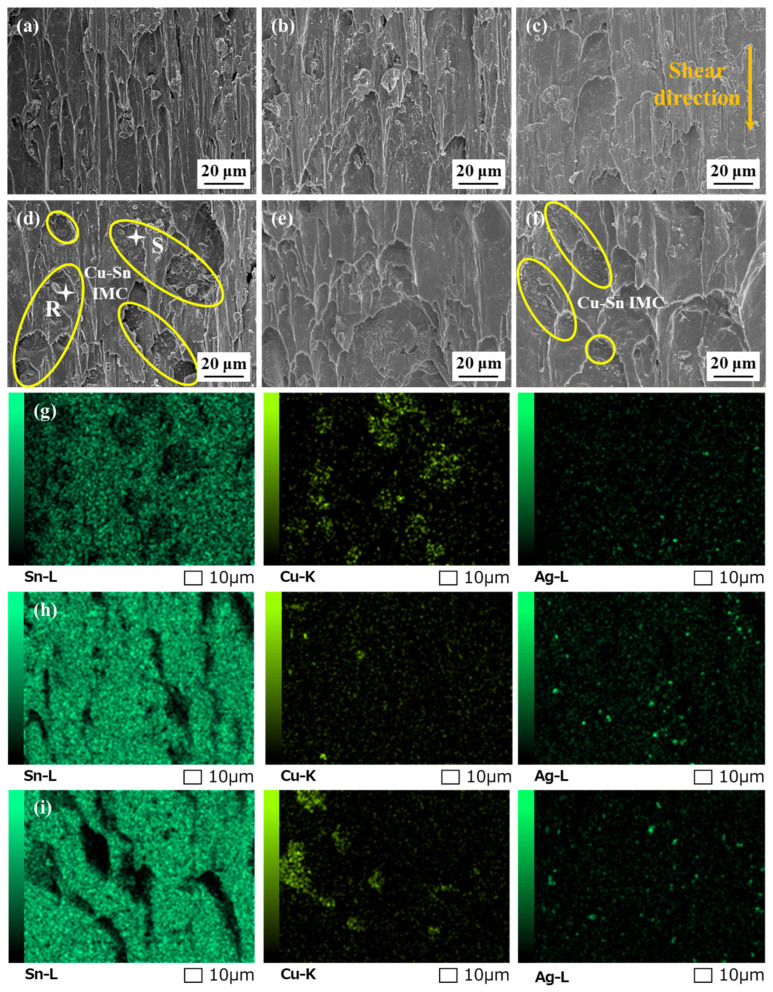
Fracture morphologies of the solder joints after (**a**–**c**) 500 cycles and (**d**–**f**) 1000 cycles: (**a**,**d**) original SAC305; (**b**,**e**) SAC305E-8; (**c**,**f**) SAC305E-12; (**g**–**i**) EDS mapping results of (**d**–**f**).

After the shear test, the fracture surfaces were examined to assess the impact of alternating thermal stress on the fracture behavior of the solder joints. Figure 17 displays the fracture morphologies of the solder joints after the thermal cycling test. After 500 cycles, as shown in Figure 17a–c, the fracture locations of all solder joints were within the solder bulk, accompanied by prominent shear dimples oriented parallel to the shear direction. Generally, in lead-free solder joints, compared with the brittle interfacial IMC layer, Sn-based solder bulk with fine plasticity displays lower strength. Therefore, cracks are more likely to initiate and propagate within the solder bulk under a low strain rate, resulting in ductile fracture of the solder joints [46]. After 500 cycles, under the action of periodic thermal load, the stress concentration level in the area near the interface layer of all solder joints was not severe, so the fracture still occurred in the solder bulk with low strength. At this time, the coarsening of the microstructure of the solder bulk was the main reason for the deterioration of the mechanical properties of all solder joints.

After 1000 cycles, the fracture surface of the original SAC305 solder joint exhibited a rough appearance, and distinctive particles were observed surrounded by the solder bulk, as shown in Figure 17d. According to the EDS mapping results in Figure 17g, the regions where these particles were situated were identified as Cu-rich areas. Furthermore, the atomic ratio of Cu to Sn in these particles closely matched 6:5 (such as Point R and S), corresponding to the composition of Cu_6_Sn_5_ interfacial IMC. Microstructural analysis revealed numerous cracks occurred within the solder bulk near the chip resistor and the Cu pad in the original SAC305 solder joint after the thermal cycling test. Under external stress, these cracks may propagate rapidly and pass through the interface between the solder bulk and Cu-Sn IMC, where severe stress concentration exists due to the significant CTE mismatch. Therefore, the fracture morphology of the original SAC305 solder joint exhibited solder bulk/interfacial IMC mixed fracture characteristic and the fracture mode transferred from ductile fracture to ductile/brittle mixed fracture. It indicated the fracture toughness of the original SAC305 solder joint was obviously decreased after undergoing long-term thermal cycling. Sun et al. [35] observed the shear fracture of Sn1.0Ag0.5Cu and Sn1.0Ag0.5Cu-0.1Al solder joints after 1500 thermal cycles and also found a similar phenomenon. During the thermal cycling test, the coarsened brittle IMC gradually became a stress concentration area and was exposed in the shear dimple after the shear test.

Nevertheless, as displayed in Figure 17e,f, the fracture morphologies of SAC305E-8 and SAC305E-12 solder joints after 1000 cycles still presented distinct dimple characteristics, showing their superior ductility compared to the original SAC305 solder joint. This behavior can be attributed to the effective release of thermal stress by the epoxy layer during the thermal cycling test. The EDS elemental mapping result in Figure 17h showed that the fracture surface of the SAC305E-8 solder joint, after 1000 cycles, primarily comprised Sn elements, revealing that its fracture location remained within the solder bulk. This indicated that the strength of the interfacial layer of the SAC305E-8 solder joint was still higher than that of the solder bulk, and cracks still preferentially initiated and propagated in the solder bulk. However, it is noteworthy that at the bottom of some shear dimples in Figure 17f, Cu_6_Sn_5_ particles were also observed, indicating the fracture path of the SAC305E-12 solder joint after 1000 cycles also partly occurred near the Cu_6_Sn_5_ layer. Considering the presence of void defects and epoxy residue in the SAC305E-12 solder joint, stress concentration was more likely to occur at the interface, leading to a ductile/brittle mixed fracture mode. However, as shown in Figure 7 and Figure 8, after 1000 cycles, no crack defect was detected in the SAC305E-12 solder joint, indicating that the accumulated thermal stress and strain were insufficient to cause crack initiation and propagation. This indicated that the cured epoxy layer on the surface of the SAC305E-12 solder joint could release stress concentration to a certain extent even if excessive epoxy was added. In summary, in this work, the SAC305E-8 solder joint exhibited the highest thermal stability during the thermal cycling test.

## 4. Conclusions

In this work, the evolution of microstructure, interfacial morphologies, and shear behaviors of epoxy-enhanced SAC305 solder joints during thermal cycling were investigated, and the influence mechanism of epoxy addition was elucidated. After 1000 thermal cycles, numerous cracks initiated and propagated within the original SAC305 solder joint, attributed to the accelerated thermal stress due to the CTE mismatch. The epoxy curing layer on the surface of SAC305E solder joints could effectively release the thermal stress and thus fewer cracks were observed within these solder joints. In addition, due to the flatter as-reflowed IMC morphologies and lower thermal stress, SAC305E solder joints exhibited smaller thicknesses of the IMC layer and finer IMC grain during the thermal cycling test. SAC305E solder joints exhibited higher shear performance and fracture toughness, which could be attributed not only to their thinner interfacial layer but also to the stress release and mechanical reinforcement effects provided by the cured epoxy layer. 

The above results indicated that the joint soldered by epoxy-enhanced SAC305 solder paste has excellent thermal cycling reliability, which is expected to be widely applied in fields such as consumer electronics and the automotive industry that require high thermal reliability of packaging materials. As electronic devices are inevitably subjected to impact or drop load during service, it is necessary to characterize the anti-drop performance of novel epoxy-enhanced SAC305 solder joints to provide a theoretical basis for developing high-performance packaging materials.

## Figures and Tables

**Table 2:** Enhancement effect of some composite solder joints bearing NPs or epoxy.

Solder Joint	Additive	Thermal Cycle Test Conditions	Enhancement Effect	Reference
SAC305	Mo NPs	−40~125 °C, 2000 cycles	Tensile strength improved by 12.4%	[18]
Sn1.0Ag0.5Cu	Al NPs	−55 to 125 °C, 1500 cycles	Shear performance improved by 29.5%	[35]
Sn1.0Ag0.5Cu	TiO_2_ NPs	−55 to 125 °C, 1500 cycles	Shear performance improved by 30.2%	[45]
Sn3.5Ag0.7Cu	Ni-coated carbon nanotubes	−40~125 °C, 2000 cycles	Shear performance improved by 44.0%	[38]
SAC305	Epoxy	−40~125 °C, 1000 cycles	Shear performance improved by 31.5%	This work

## Data Availability

The original contributions presented in the study are included in the article, further inquiries can be directed to the corresponding author.
